# Glioblastoma Cells Express and Secrete Alternatively Spliced Transcripts of Coagulation Factor X

**DOI:** 10.3390/biomedicines13030576

**Published:** 2025-02-25

**Authors:** Xiaotian Li, Xilei Liu, Yalong Gao, Lei Li, Yajuan Wang, Jianlong Men, Jing Ren, Jiwei Wang, Fanjian Li, Yaohua Li, Jianhua Xiong, Xiaoteng Cui, Cheng Wei, Cong Wang, Jingfei Dong, Li Liu, Jianning Zhang, Shu Zhang

**Affiliations:** 1 Department of Neurosurgery, Tianjin Medical University General Hospital, Tianjin 300052, China; xiaotianli@tmu.edu.cn (X.L.); yalong_gao@tmu.edu.cn (Y.G.); lilei3602@163.com (L.L.); fanjianli1994@hotmail.com (F.L.); liyaohua5@163.com (Y.L.); doctorxiong@hotmail.com (J.X.); xiaotengcui@tmu.edu.cn (X.C.); mhxyweicheng@163.com (C.W.); wangcong@tmu.edu.cn (C.W.); 2 Key Laboratory of Post-Neuro-Injury Repair and Regeneration in Central Nervous System, Tianjin Neurological Institute, Tianjin 300052, China; 13132522782@163.com; 3 Department of Urology, Tianjin Medical University General Hospital, Tianjin 300052, China; liuxilei@tmu.edu.cn; 4 Department of Gynecology and Obstetrics, Tianjin Medical University General Hospital, Tianjin 300052, China; wangyajuan95@tmu.edu.cn; 5 Center for Precision Medicine, Tianjin Medical University General Hospital, Tianjin 300052, China; thromhaemo@126.com (J.M.); renjingzyy@126.com (J.R.); 6 Department of Neurosurgery, Tianjin Huanhu Hospital, Tianjin 300350, China; wangjiwei@tmu.edu.cn; 7 Bloodworks Research Institute, Division of Hematology, Department of Medicine, University of Washington School of Medicine, Seattle, WA 98195, USA; jfdong@psbc.org

**Keywords:** coagulation factor X, glioblastoma, hypercoagulability, thrombosis, splice transcripts

## Abstract

**Background:** Patients with cancer often develop a prothrombotic state that can evolve into venous and arterial thrombosis, which is associated with poor clinical outcomes. Glioblastoma multiforme (GBM) is the most frequently associated with thrombosis, but the underlying causes of this prothrombotic state are poorly defined. **Objectives:** We designed a study to characterize the expression of coagulation factor X (FX) and its alternatively spliced transcripts in glioblastoma tissues surgically removed from patients and in clonal cell lines. **Methods:** The *F10* mRNA and FX protein were quantified in tissues surgically removed from seven patients with glioblastoma (glioma grade 3–4) and those from non-tumor patients. Glioblastoma cells from three human clonal lines were examined for the expression and secretion of FX at baseline and after the cells were stimulated with lipopolysaccharide (LPS) or subjected to oxygen/glucose starvation in culture. PCR products were subjected to Sanger sequencing and amplicon sequencing to identify *F10* isoforms and their ratios. A chromogenic assay was performed to assess FX activity. **Results:** Glioblastoma tissue and cell lines expressed high levels of the full-length and an alternatively spliced *F10* mRNA. The latter produced a C-terminal truncated FX. The ratio of full-length to truncated *F10* transcripts was significantly higher in normal brain tissues than in glioblastoma tissue. In cultured cells from the glioblastoma cell lines, FX was secreted to the conditioned medium and was active in cleaving a chemical substrate. The FX expression and secretion were upregulated in cells stimulated with LPS or subjected to oxygen/glucose starvation. **Discussion**: Glioblastoma cells synthesize and secrete FX that was active in promoting thrombin generation. These findings provide a new underlying mechanism to explain why glioblastoma patients are prone to developing thrombosis.

## 1. Introduction

Venous thromboembolism (VTE) is frequently associated with brain tumors [1,2]. Approximately 30% of patients with glioblastoma multiforme (GBM, which is a poorly differentiated glioma) develop VTE, at rates significantly higher than those with well-differentiated low-grade gliomas [3]. Patients with GBM often develop dysregulated coagulation and fibrinolysis, as well as high levels of platelet activation [4]. For example, GBM patients with elevated activity of coagulation factor VIII increase the risk of VTE by 2.1-fold [5]. Brain tumor patients had an increased risk of VTE when the plasminogen activating system was blocked [6]. Although the association between GBM and thrombosis has been demonstrated in extensive clinical observational studies, the underlying cause of the GBM-induced systemic prothrombotic state and thrombosis remains poorly defined, especially given the fact that GBM cells survive poorly in systemic circulation.

Coagulation factors are primarily synthesized in and released from hepatocytes in the liver [7,8,9,10,11,12], but some also originate from other cells [13,14,15,16,17,18]. Here, we report results from a study designed to characterize GBM cell-derived FX and its mRNA transcripts for activating coagulation. The findings provide new insight into the underlying etiology of the brain tumor-associated prothrombotic state and thrombosis.

## 2. Materials and Methods

### 2.1. Reagents

The primary antibodies used for immunoblots and fluorescence immunohistochemistry were (1) a monoclonal antibody against human GAPDH (ab8245; Abcam, Cambridge, UK), (2) a rabbit polyclonal antibody against human FX (ab180701; Abcam), (3) a sheep anti-human FX antibody (PAHFX-S; Haematologic Technologies, Essex Junction, VT, USA), (4) a rabbit polyclonal antibody against both human and mouse activated FXa (PA5-29118; Thermo Fisher Scientific, Waltham, MA, USA), (5) a mouse antibody against NeuN (ab104224; Abcam), which is a common marker for neurons, and (6) a rabbit antibody against human glial fibrillary acidic protein (GFAP) (ab7260; Abcam), which served as a marker for glial cells. The secondary antibodies used for fluorescent immunohistochemistry included donkey AlexaFluor-488 anti-sheep IgG (A-11015; Thermo Fisher Scientific), donkey AlexaFluor-555 anti-rabbit IgG (A-31572; Thermo Fisher Scientific), and donkey AlexaFluor-555 anti-mouse IgG (A-31570; Thermo Fisher Scientific). Recombinant human FX was purchased from Sino Biological (11076-H08B; Beijing, China). LPS was purchased from Macklin (L861706; Ghaziabad, India). TaqMan probes were purchased from Sangon Biotech (Shanghai, China). The protein markers for immunoblots (PageRuler) were purchased from Thermo Scientific (#26616).

### 2.2. Tissue Samples from Patients

Tumor tissue samples were collected from surgically removed glioblastomas from 7 patients. Cerebral tissue from patients with traumatic brain injury or intracerebral hemorrhage undergoing decompressive craniectomy was collected as normal brain tissue controls (*n* = 4). Upon collection, the tissue samples were washed extensively and cryosectioned into 8 µm slices for fluorescent immunohistochemistry. Informed consent was obtained from all patients and control subjects or their guardians. This study was approved by the Joint Ethics Committee of Tianjin Medical University General Hospital (approval IRB2024-KY-033).

### 2.3. Cell Culture

Cells from the three human glioblastoma cell lines, U87, U251, and SNB19 (American Type Culture Collection, Manassas, VA, USA), were maintained in Dulbecco’s Modified Eagle Medium (DMEM; C11995500BT; Thermo Fisher Scientific) supplemented with 10% fetal bovine serum (10099141C; Thermo Fisher Scientific). The human hepatic HepG2 cells were cultured as positive controls. The conditioned medium was analyzed for FX.

A subset of cultured cells were stimulated with LPS at 1, 10, 100, or 1000 ng/mL for 24 h at 37 °C to determine whether (1) an inflammatory mediator changed the expression of *F10 mRNA* and FX protein because inflammation consistently develops in cancer patients and (2) FX is stored in intracellular granules and released from them upon stimulation. In addition, the glioblastoma U87 cells were also grown in complete growth medium (95% O_2_ and 5% CO_2_ at 37 °C) to 70–80% of confluence. They were then washed with PBS and incubated with serum-free growth medium for 24 h followed by incubation in the serum- and glucose-free DMEM at an atmosphere of 1% O_2_, 5% CO_2_, and 94% N_2_ for 2, 4, 6, or 8 h at 37 °C. This condition of oxygen and glucose starvation was designed to mimic the microenvironment of glioblastoma growth [19]. Control cells were cultured under the condition of 95% O_2_ and 5% CO_2_ at 37 °C in the presence of glucose. The conditioned medium was then collected, and proteins and mRNA were extracted from the treated cells for further testing.

### 2.4. RNA Interference and Lentivirus Infection

The target sequences of *F10* short hairpin RNAs (shRNAs) used for RNA interference were as follows: 5′-GCACCTGTTTAGAAGGATTCG-3′ (sh-FX-1), 5′-GCATCACATGGAAGCCATATG-3′ (sh-FX-2), 5′-GCAGCTTCATCATCACCCAGA-3′ (sh-FX-3), and 5′-TTCTCCGAACGTGTCACGT-3′ (sh-NC). These were synthesized by a commercial vendor (Shanghai Integrated Biotech Solutions, Shanghai, China) and inserted into a pGM–lentivirus vector carrying a GFP reporter. For viral transfection, U87 and SNB19 cells were plated in 6-well plates at 3 × 10^5^ cells/mL and allowed to grow to 20% confluence. They were then incubated with individual shRNA lentivirus (multiplicity of infection = 5) for 48 h and examined for the transfection efficiency by detecting intracellular GFP under a fluorescence microscope (Olympus Corp., Tokyo, Japan). Seventy-two hours after lentivirus infection, puromycin (2 μg/mL; A1113803; Thermo Fisher Scientific) was added. Cells were harvested after 7 days in culture to determine the gene knockdown efficiency using immunoblots and quantitative PCR (RT-qPCR).

### 2.5. Immunoblots

Human glioblastoma cells were lysed in RIPA buffer (R0010; Solarbio, Beijing, China) in the presence of a protease inhibitor cocktail and phosphatase inhibitors for 30 min on ice. The cell lysates were centrifuged at 12,000 rpm for 10 min at 4 °C to collect the supernatant, which was boiled for 10 min. The total protein concentration was measured in each sample and standardized to 20 µg of total proteins per well before the samples were subjected to 10% sodium dodecyl sulfate–polyacrylamide gel electrophoresis (SDS-PAGE), and transferred to a polyvinylidene fluoride (PVDF) microporous membrane. The membrane was blocked with 5% milk in Tris-buffered saline containing Triton X-100 (TBST) for 1 h at 25 °C, followed by incubation with TBST with either an anti-FX or an anti-GAPDH antibody overnight at 4 °C. After washing, the membrane was incubated with an HRP-conjugated anti-rabbit secondary antibody for 1 h at 25 °C. The antibody binding was visualized using an enhanced chemiluminescence assay (ECL; 34580; Thermo Fisher Scientific) and quantified by densitometry using the Image J 1.41 program.

### 2.6. Quantitative Real-Time PCR

Glioblastoma cells were washed with PBS and lysed using TRIzol (Thermo Fisher Scientific). The total mRNA was extracted with chloroform, precipitated with isopropanol, and quantified using a NanoDrop (Thermo Fisher Scientific). RNA (2 μg) from each sample was reversely transcribed into cDNA according to the manufacturer’s instructions (FSK-101; Toyobo, Osaka, Japan). The forward and reverse primers for the *F10* mRNA were 5′-ACATCCTGGCGAGGGTCA-3′ and 5′-CGAGGCCGTCTTTACATTTG-3′. *Gapdh* was used as a control and amplified using the following primers: 5′-GAAGGTGAAGGTCGGAGTC-3′ and 5′-GAAGATGGTGATGGGATTTC-3′ (Sangon Biotech). The cDNA (200 ng) was amplified in triplicate using RT-PCR under the following conditions: 50 °C for 2 min; 95 °C for 2 min; 40 cycles of 15 s at 95 °C, 15 s at 60 °C, and 60 s at 72 °C; and 72 °C for 60 s, according to the manufacturer’s instructions (Thermo Fisher Scientific). The results were analyzed using the comparative threshold cycle (Ct) method and normalized to the expression level of the reference gene *gapdh*. The relative expression of the target mRNA was quantified using the 2^−ΔΔCT^ method.

### 2.7. Sanger Sequencing of F10 Transcripts

Double-stranded cDNA was synthesized using a ReverTra Ace-α cDNA Synthesis Kit (FSK-101; Toyobo). The *F10* gene was amplified via PCR using 2 × Hieff NGS HG Multiplex PCR Master Mix (13283ES03; Yeasen Biotechnology, Shanghai, China) and gene-specific primers for *F10*: forward, 5′-GGACACAGTACTCGGCCACACCAT-3′; reverse, 5′-AATGGAGAGGACGTTATGACC-3′. The PCR products were cloned into a pUC19 vector using a pMD 18-T Vector Cloning Kit (6011; Takara Bio Inc., Kyoto, Japan). The cDNA sequences of *F10* transcripts were detected and confirmed using Sanger sequencing by a commercial service (Genewiz, Suzhou, Jiangsu, China). The results were analyzed for confirmation and alignment using the National Center for Biotechnology’s Information BLAST-2.14.0 tool.

### 2.8. Amplicon Sequencing

Amplicon sequencing was performed to estimate the ratio of *F10* gene transcripts. Specifically, double-stranded cDNA was synthesized using a ReverTra Ace-α cDNA Synthesis Kit (FSK-101; Toyobo), and the target fragments were amplified from the cDNA using specific primers with different barcodes. PCR was performed using HiFi PCR SuperMix (AS131-01; TransGen Biotech Co., Ltd., Beijing, China), and the products were purified with the EasyPure PCR Purification Kit (EP101-01; TransGen). The PCR products were separated on a 2% agarose gel and analyzed using high-throughput sequencing (Genewiz). Bioinformatics analysis was performed to determine the ratio of the *F10* transcripts.

### 2.9. Fluorescent Immunohistochemistry

Blocks of glioblastoma and control brain tissues were fixed in 4% paraformaldehyde (P1110; Solarbio) for 24 h at 4 °C and immersed in a sucrose gradient (15% and 30%). They were then embedded in a cutting compound (4583; Sakura Finetek USA, Inc. Torrance, CA, USA), frozen in liquid nitrogen, and sectioned. The sections were blocked for non-specific binding and permeabilized with PBS containing 3% bovine serum albumin (BSA), 5% normal donkey serum, and 0.3% Triton X-100 for 90 min at room temperature. The sections were then incubated with sheep anti-FX, rabbit anti-GFAP (1:200), or mouse anti-NeuN antibodies overnight at 4 °C, followed by washing with PBS and incubation with secondary antibodies (AlexaFluor-488 donkey anti-sheep IgG, AlexaFluor-555 donkey anti-rabbit IgG, or AlexaFluor-555 donkey anti-mouse IgG) for 60 min at room temperature. After washing with PBS, the nuclei were stained with DAPI. The sections were examined under a fluorescence microscope (IX73; Olympus Corp., Tokyo, Japan) at 488 nm excitation and at 555 nm emission. In addition to tissue section, cells grown on poly-D-lysine-coated coverslips were washed in ice-cold PBS and fixed for 10 min in 4% formaldehyde at room temperature. After blocking and permeabilization in PBS containing 3% BSA and 0.3% Triton X-100 for 90 min, the cells were stained with antibodies against FX, GFAP, and NeuN, separately.

### 2.10. FX Enzyme-Linked Immunosorbent Assay

Confluent U87 cells in culture were washed with PBS and stimulated with 0, 1, 10, 100, or 1000 ng/mL of LPS for 24 h to mimic cancer-associated inflammation. The supernatant was then collected, centrifuged at 1500 rpm at room temperature for 10 min to remove cell debris, and analyzed for FX using a Human FX ELISA Kit (ab108832; Abcam).

### 2.11. FX Activity

FX activity was detected using N-benzoyl-l-isoleucyl-l-glutamyl-glycyl-l-arginine-p-nitroaniline hydrochloride as the substrate in a chromogenic assay (S2222; Diapharma, West Chester Township, OH, USA), as previously described [18]. We chose this assay because it is a fully automated clinical assay and has been widely used in previous studies [20]. In brief, cells were grown in T-75 flasks in the complete medium with 10% fetal bovine serum until 100% confluence. They were then washed with PBS and incubated with 1 mL of 50 mM Tris buffer (pH 7.4) containing 1% BSA and 5 mM Ca^2+^ and 500 µL of the chromogenic substrate for 5 h at 37 °C. The supernatant (100 µL) was collected every hour and analyzed for cleaving of the substrate using the BioTek Synergy HT Microplate Reader (BioTek Instruments, Winooski, VT, USA).

In addition to the chemical substrate, we also performed a clotting assay to measure the ability of GBM-derived extracellular vesicles (GBM-EVs) to promote coagulation. We chose to test EVs instead of glioblastoma cells because patients with GBM often develop a systemic prothrombotic state or extracranial thrombosis [21,22], but GBM cells can not survive in the systemic circulation [23,24,25]. In contrast, EVs from glioblastoma cells are detected at significant levels in these patients [26,27]. For this assay, EVs were enriched from lysates of glioblastoma (GBM-EVs) and normal cerebral tissues (NBT-EVs) by centrifuging tissue lysates at 12,000× *g* for 5 min at 4 °C to collect the supernatant, which was then centrifuged at 100,000× *g* for 60 min at 4 °C (twice) to collect the pellets. The GBM-EVs and NBT-EVs (10^4^/µL) were mixed with 200 µL of the FX-deficient plasma and 170 μL of 20 mM calcium chloride (CaCl_2_) at 37 °C. Clotting time was then measured using Century Clot analyzer (Century Yikang Medical Technology Development Co., Ltd., Tianjin, China). We chose to test EVs from GBM tissue, not from plasma samples from patients, because plasma contains EVs from GBM tissue, endothelial cells, and blood cells.

### 2.12. Statistical Analysis

The results were reported as mean ± standard deviation and were analyzed using Student’s *t*-test for comparison between two groups and one-way ANOVA for multiple group comparisons after the data passed the normality test using SPSS for Windows (v22.0; IBM Corp., Armonk, NY, USA). The data that failed the normality test were analyzed by the Mann–Whitney rank sum and one-way ANOVA on ranks tests. Two-way ANOVA was used to compare levels of FX activity on the surface of glioblastoma cells. A *p*-value of <0.05 was considered statistically significant.

## 3. Results

### 3.1. FX Expression in Human Glioblastoma Tissue and Clonal Cells

**Figure 1:**
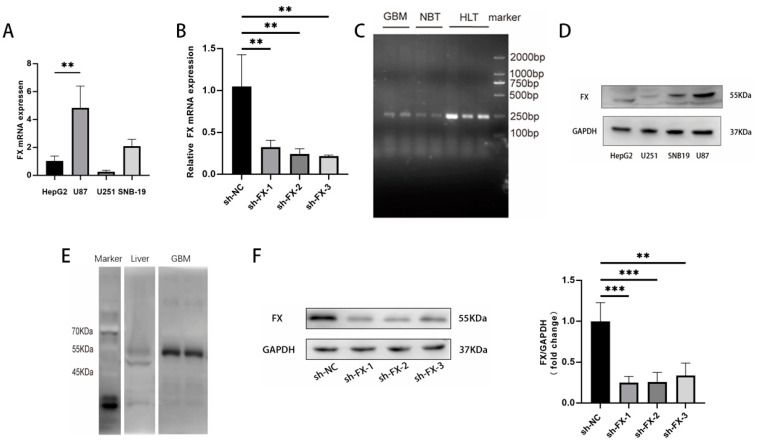
Expression of FX in glioma tissues and cell lines. (**A**) *F10* mRNA levels in cells from the GBM cell lines U87, U251, and SNB-19 relative to the levels in HepG2 cell (*n* = 3, one-way ANOVA). HepG2 cells from the liver were used as a positive control. (**B**) RT-qPCR showed that *F10* mRNA levels decreased in *F10*-knockdown U87 cells (sh-*F10*-1, sh-*F10*-2, sh-*F10*-3, *n* = 3 for each shRNA construct). (**C**) Detection of *F10* transcripts in GBM specimens, healthy brain tissue (NBT: normal brain tissue), and human liver tissue (HLT: human liver tissue). Representative immunoblots show (**D**) FX protein expressed in cells from the three clonal glioblastoma lines of U251, SNB19, and U87 and (**E**) FX in glioblastoma tissue samples. HepG2 cells were tested as positive controls. (**F**) The synthesis of FX protein was suppressed in glioblastoma U87 cells sh-FX-1, sh-FX-2, and sh-FX-3 (*n* = 3 for each shRNA construct). The quantitative data were analyzed using one-way ANOVA, *** *p* < 0.001, ** *p* < 0.01.

Using RT-qPCR, we detected the *F10* mRNA in cells from three clonal human glioblastoma lines. U87 and SNB-19 cells contained 4.8 and 2.0 times as much *F10* mRNA as hepatocyte-derived HepG2 cells, but *F10* mRNA was at a residual level in U251 cells (Figure 1A). For validation, the expression of the *F10* mRNA was suppressed in U87 cells transfected with each of three complementary lentivirus inhibitory shRNAs (Figure 1B). In addition to clonal cell lines, we also detected *F10* mRNA in glioblastoma tissue from tumor masses removed from patients during surgery (Figure 1C).

FX was also detected at protein levels in cells from the clonal glioblastoma cell lines U87 and SNB19 (Figure 1D) and glioblastoma tissue samples from patients (Figure 1E) using immunoblots. FX was again minimally detected in U251 cells. FX detected in glioblastoma tissue and clonal cells appears to be smaller in molecular mass compared to FX found in HepG2 cells. The difference could be attributed to different levels of glycosylation [28,29]. The three lentivirus inhibitory shRNAs suppressed the expression of FX in U87 cells by 74.9% ± 6.1%, 74.3% ± 9.5%, and 66.3% ± 12.3%, respectively (Figure 1F). These results demonstrated that *F10* mRNA is transcribed and FX is synthesized in human glioblastoma tissue and derivative clonal cell lines.

### 3.2. Upregulation of FX in Cells Deprived of Oxygen and Glucose

**Figure 2:**
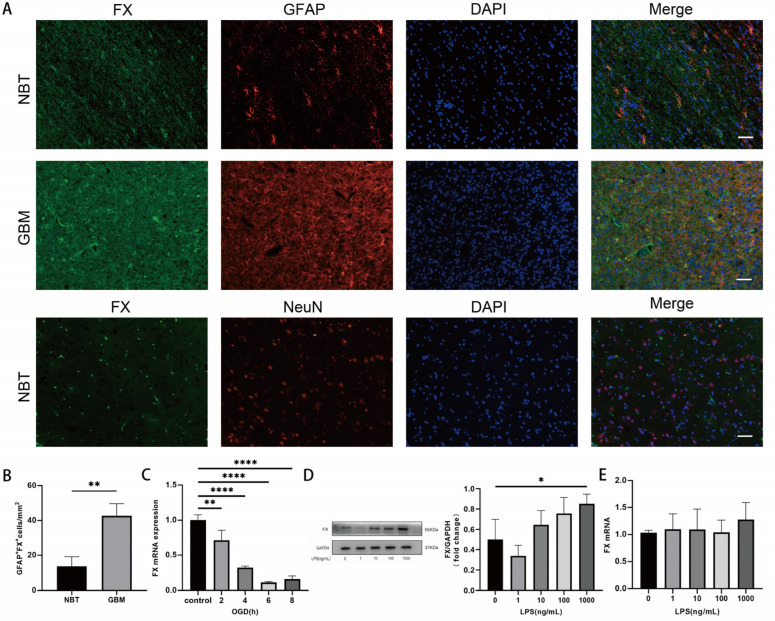
Regulation of FX protein expression. (**A**) GBM and normal brain tissue (NBT) were sectioned and probed for FX in neurons identified by NeuN and glial cells identified by GFAP using specific antibodies (bar = 50 μm) and (**B**) the overall expression of FX in these tissues was quantified by fluorescence intensity using an image scan (*n* = 3, *t*-test). (**C**) U87 cells were cultured in the condition of hypoxia and serum starvation (i.e., OGD: oxygen and glucose depletion) for different times at 37 °C. They were then processed for quantifying *F10* mRNA (*n* = 3, one-way ANOVA). (**D**) Cultured U87 cells were stimulated with increasing doses of LPS for 24 h and then processed for immunoblot with an FX antibody and quantified using densitometry (left panel: a representative immunoblot image, right panel: a summary of 3 independent experiments (one-way ANOVA). (**E**) RT-qPCR was performed to quantify levels of the *F10* mRNA in U87 cells after stimulation with LPS (*n* = 3, one-way ANOVA). For all figures, * *p* < 0.05, ** *p* < 0.01, **** *p* < 0.0001.

Using immunohistopathology, we also detected FX in glial cells of glioblastoma tissue identified by the glial cell marker GFAP (middle panel, Figure 2A). In contrast, FX was not detected in neurons identified by the neuronal marker NeuN (bottom panel, Figure 2A). The levels of FX staining were quantitatively greater in glioblastoma tissues than in non-cancerous cerebral tissue, defined by the number of FX^+^ cells (Figure 2B). More importantly, the expression of *F10* mRNA was progressively reduced in U87 cells that were cultured over time in the condition of oxygen and glucose deprivation (Figure 2C), which is often found in the microenvironment of the glioblastoma tissue niche [30,31,32]. In contrast, U87 cells increased their FX expression when they were exposed to 1000 ng/mL of the endotoxin LPS (Figure 2D), without a significant change to the levels of *F10* mRNA (Figure 2E). LPS was tested to mimic the condition of inflammation and severe infection, both of which were associated with increased risk of thrombosis in patients with glioblastoma [33], and to determine that an inflammatory-like condition induced by LPS did not affect the rate of FX synthesis, and that newly synthesized FX is not stored significantly in intracellular storage granules, but is constitutively released. These results demonstrated that FX synthesis in glioblastoma cells dynamically responds to changes in the local cancer niche and systemic circulation.

### 3.3. Release and Coagulant Activity of FX from Glioblastoma Cells

**Figure 3:**
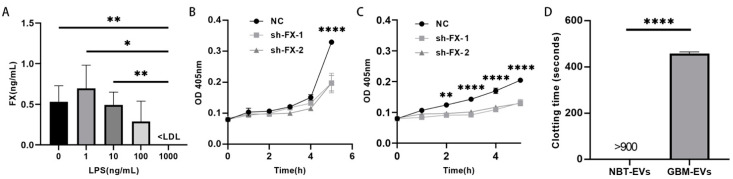
FX released from cultured GBM cells was active. (**A**) FX was released into the conditioned medium of cultured GBM U87 cells measured using ELISA, and its release was affected by the LPS stimulation (*n* = 3). (**B**) FX in the conditioned medium from cultured U87 (**B**) and SBN-19 (**C**) cells cleaved the chromogenic substrate S-2222 (*n* = 10, two-way ANOVA, * *p* < 0.05, ** *p* < 0.01, **** *p* < 0.0001). (**D**) Clotting time induced in FX-deficient plasma by EVs from human glioblastoma and normal brain tissue (*n* = 3, one-way ANOVA, **** *p* < 0.0001).

In addition, we also detected FX in the conditioned medium collected from cultured U87 cells at the baseline condition, but the FX release was progressively reduced when the cells were treated with increasing concentrations of LPS for 24 h at 37 °C (Figure 3A). FX released from cultured U87 (Figure 3B) and SNB-19 cells (Figure 3C) was able to cleave the chromogenic substrate S-2222. Because this chemical substrate is not specific to FXa activity, we also measured the ability of EVs from glioblastoma cells to induce coagulation in the clotting time, which is commonly used for the clinical evaluation of a systemic prothrombotic state in patients with cancer [34,35]. The glioblastoma-derived EVs induced significant clot formation in the FX-depleted plasma, resulting in a drastically shortened clotting time (Figure 3D). We chose to test glioblastoma-derived EVs instead of plasma from these patients to detect membrane-bound FX for clotting because glioblastoma cells survive poorly in the systemic circulation [25], but patients with the cancer often develop a systemic prothrombotic state [21], which is likely triggered by circulating mediators. Glioblastoma-derived EVs likely serve as the mediators to disseminate a systemic prothrombotic state and contribute to resultant thrombosis in the absence of either glioblastoma masses or individual cells. These results demonstrate that FX is released from glioblastoma cells, and its release is dynamic depending on the ischemic and inflammatory states in the cancer microenvironment.

### 3.4. F10 Transcripts and Ratio in Glioblastoma

**Figure 4:**
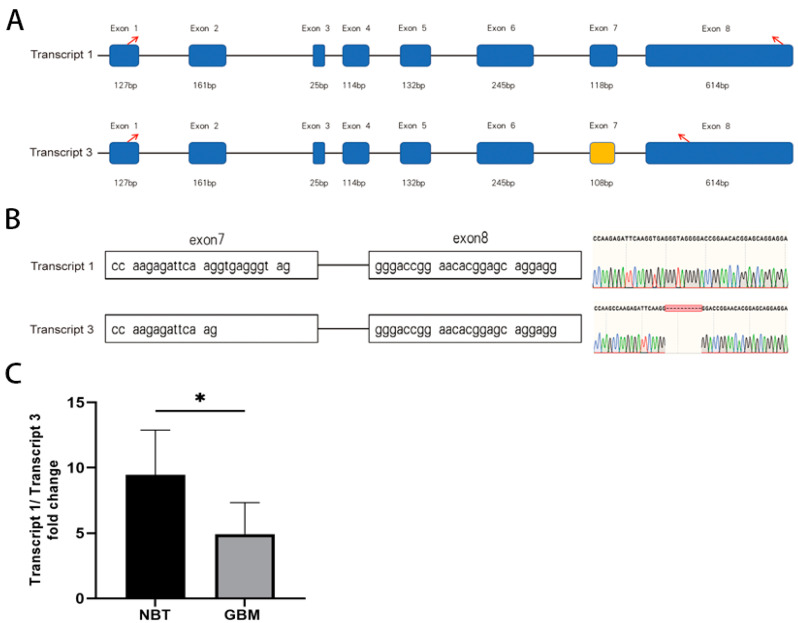
*F10* transcript analysis. (**A**) Coding exons included in the different isoform mRNAs of *F10*. Blue boxes represent exons. Due to the missing nucleotide sequence in exon 7 (yellow), translation was terminated prematurely when it reached exon 8 (red arrow moved forward). (**B**) Variants identified by Snapgene: *F10* mRNA transcripts were cloned from healthy human brain tissue and glioblastoma tissue using TA cloning. The base deletion in exon 7 of the mRNA transcript 3 is shown in the lower panel, and the upper panel shows bases at the same position in transcript 1. (**C**) The ratios of the two variants in 4 normal healthy brain samples and 7 glioblastoma samples. * *p* < 0.05.

Using reverse transcription and amplification of cDNA followed by TA cloning and Sanger sequencing, we detected two transcripts of the *F10* gene in glioblastoma tissue samples from seven patients: transcript 1 was the full length of the human *F10* gene (top panel, Figure 4A), and transcript 3 was found with exon 7 spliced (bottom panel, Figure 4A), resulting in a novel exon 7 with a deletion of 10 nucleotides (Figure 4B). Both full-length and truncated *F10* transcripts were also detected in non-cancerous cerebral tissue, but the transcript 1-to-transcript 3 ratio was significantly higher in non-cancerous cerebral tissues than in glioblastoma tissues (Figure 4C). These results demonstrate the presence of the full-length *F10* and a truncated *F10* variant (transcript 3), and the truncated *F10* is predominately found in glioblastoma cells.

## 4. Discussion

In this study, we presented several lines of experimental evidence that FX was transcribed, synthesized, and released from glial cells and glial cell-derived glioblastoma cells. We detected a new truncated *F10* variant by alternative splicing, and it was predominantly expressed in glioblastoma cells. We also showed that cells subjected to hypoxic conditions and serum starvation or challenged with proinflammatory LPS changed the expression and release of FX. We made the following novel observations.

First, although it was originally detected in hepatocytes of the liver [7], FX has recently been detected in human endothelial cells [13,14,15,16] and in tumor-associated monocytes and macrophages [36,37]. Here, we report the detection of *F10* mRNA and FX protein in glioblastoma tissues that have been surgically removed from patients and in cells from clonal glioblastoma cell lines. The results from this study are supported by several early reports. First, the *F10* mRNA has been detected in rat brain tissue, but its cellular location is not reported [38]. Furthermore, Liu et al. reported the hypomethylation of the *F10* gene in most glioma specimens [39], without further determining whether the *F10* gene is fully transcribed to synthesized FX. An important observation was that cells from three clonal glioblastoma lines expressed different levels of the *F10* mRNA. The exact reason for this differential expression of FX remains to be investigated, but it could be attributed to the phenotypical differences among these cells at multiple levels. For example, the high FX-expressing U87 cells have a neuronal-like phenotype and a high rate of proliferation, whereas the U251 cells, which expressed residual FX, have a mesenchymal-like phenotype and a low rate of proliferation [40], suggesting that FX expression is developmentally regulated in glial cells. However, the single nucleotide polymorphism array analysis indicates that SNB-19 and U251 cells are developed from the same patient [41], suggesting that in vitro transformation of original cancer cells also affects the *F10* transcription and FX expression. More importantly, we showed for the first time the predominant expression of a truncated *F10* gene transcript in human glioblastoma tissue and cells.

Second, FX was constitutively synthesized and secreted from cultured glioblastoma cells, but its intracellular quantity decreased in U87 cells subjected to hypoxia and serum starvation but increased in those stimulated with a high dose of LPS. However, we can not exclude the possibility that the FX reduction in cells subjected to hypoxia and serum starvation is attributable to increasing cell death, as glioblastoma masses often develop severe tissue narcosis due to tissue ischemia. Nevertheless, these results were important because the two treatments mimic the microenvironment of the glioblastoma tissue niche and the systemic inflammatory state found in patients with glioblastoma, respectively.

Third, we demonstrate that FX secreted from glioblastoma cells was constitutively active in cleaving an FX substrate in the presence of calcium, and glioblastoma-derived EVs promoted coagulation in vitro. This observation is important because circulating FX in healthy subjects is a zymogene, so it is active when cleaved by either activated factor IXa in the presence of factor VIIIa (intrinsic pathway) or by the tissue factor–factor VIIa complex generated through the extrinsic coagulation pathway. Furthermore, we demonstrate for the first time that glioblastoma cells and normal brain glial cells express an alternatively spliced and truncated *F10*. Although we were unable to quantify the levels of FX synthesized and released from glioblastoma and normal glial cells because of a limited sample size, we show that the truncated *F10* is predominantly expressed in glioblastoma cells. Because the truncation occurs in the C-terminus, it is unlikely to be why glioblastoma cells constitutively release FX. Furthermore, the finding that glioblastoma-derived EVs promote coagulation strongly demonstrates that FX is membrane-bound.

This study was limited to investigating the differential coagulation activities of FXs encoded by the two glial cell-derived *F10* transcripts. We also did not investigate whether the FX secreted from glioblastoma cells affects cancer growth and differentiation. How glioblastoma-derived FX is activated also requires additional investigation [42].

In summary, we have presented evidence that glial cells from the human brain and glioblastoma cells expressed and secreted two distinct transcripts that encode the full-length *F10* and a truncated variant and that the truncated variant is predominant in the glioblastoma cells. This glial cell-derived FX is enzymatically active. Our study also raises the question of whether glial cell-derived FX, especially its truncated variant, contributes to the prothrombotic state and venous thrombosis associated with glioblastoma [43,44]. Sufficiently powered clinical studies will be needed to evaluate the contribution of glioblastoma-derived FX to the prothrombotic state and thrombosis often found in patients with glioblastoma.

## Data Availability

The data presented in this study are available on request from the corresponding author.
